# Impact of SGLT2 Inhibitors on Quality of Life in Heart Failure Across the Ejection Fraction Spectrum: Systematic Review and Meta-analysis

**DOI:** 10.1016/j.cjco.2023.12.002

**Published:** 2023-12-10

**Authors:** Yaksh R. Shah, Ricky D. Turgeon

**Affiliations:** a Faculty of Pharmaceutical Sciences, University of British Columbia, Vancouver, British Columbia, Canada

## Abstract

**Background:**

Use of a sodium-glucose cotransporter-2 inhibitor (SGLT2i) reduces hospitalization in heart failure (HF) patients across the spectrum of ejection fraction, but no study has comprehensively explored their impact on quality of life (QoL) with respect to different subgroup populations. We aimed to explore the QoL impact of SGLT2i use in HF patients across the spectrum of ejection fraction and over time.

**Methods:**

We searched MEDLINE, Embase, and Cochrane Central Register of Controlled Trials (CENTRAL) covering the period from 2019 to February 2022. We included placebo-controlled randomized controlled trials (RCTs) enrolling HF patients that evaluated QoL as an outcome. Two reviewers independently assessed studies for eligibility, extracted data, and assessed risk of bias (RoB), using the Cochrane RoB2 tool, and certainty of evidence, using the Grading of Recommendations Assessment, Development and Evaluation (GRADE) framework. Primary and secondary outcomes were the mean difference in QoL, and clinically important improvement in QoL, as defined in the original study, respectively. We conducted subgroup analyses based on ejection fraction category, SGLT2i agent, and timing of QoL measurement.

**Results:**

From 1477 identified reports, we included 14 RCTs (n = 23,361). The mean age was 68 years, and 34% were female. All included RCTs reported QoL using the Kansas City Cardiomyopathy Questionnaire (KCCQ). SGLT2i use improved KCCQ-overall summary score, compared with placebo (mean difference 2.0, 95% confidence interval 1.6-2.5; high certainty). More patients receiving an SGLT2i achieved a clinically important QoL improvement (risk ratio 1.14, 95% confidence interval 1.02-1.28; moderate certainty). Similar improvements were observed in the KCCQ clinical summary and total symptom subscores, and across all subgroups and timeframes.

**Conclusions:**

Use of an SGLT2i consistently provides a clinically important improvement in QoL among patients with HF, regardless of ejection fraction, with noticeable improvements seen as early as week 2.

## RÉsumÉ

**Contexte:**

Les inhibiteurs du cotransporteur sodium-glucose de type 2 (SGLT-2) réduisent le nombre d’hospitalisations chez les patients atteints d’insuffisance cardiaque (IC), quelle que soit la fraction d’éjection. Cependant, aucune étude n’a examiné de manière exhaustive leur incidence sur la qualité de vie (QdV) dans différents sous-groupes de populations. Notre étude visait à explorer l’incidence de l’utilisation d’inhibiteurs du SGLT-2 sur la QdV des patients atteints d’IC, en fonction de la fraction d’éjection et au fil du temps.

**Méthodologie:**

Nous avons effectué des recherches dans les bases de données MEDLINE, Embase et le registre Cochrane Central Register of Controlled Trials (CENTRAL) couvrant la période de 2019 à février 2022. Nous avons inclus des essais contrôlés randomisés (ECR) contrôlés par placebo menés chez des patients atteints d’IC, dont les critères d’évaluation portaient sur la QdV. Deux examinateurs ont évalué indépendamment les critères d’admissibilité aux études, extrait les données et évalué le risque de biais, à l’aide de l’outil Cochrane RoB2, et la certitude des données probantes, à l’aide du cadre GRADE (*Grading of Recommendations Assessment, Development and Evaluation*). Les critères d’évaluation primaire et secondaire correspondaient à ceux définis dans l’étude d’origine et étaient respectivement la différence moyenne relative à la QdV et l’amélioration d’importance clinique de la QdV. Nous avons effectué des analyses par sous-groupes portant sur la catégorie de fraction d’éjection, l’inhibiteur du SGLT-2 utilisé et le moment de la mesure de la QdV.

**Résultats:**

Sur les 1 477 rapports recensés, nous avons inclus 14 ECR (n = 23 361). L’âge moyen était de 68 ans, et 34 % étaient des femmes. Tous les ECR inclus ont rapporté la QdV à l’aide du questionnaire KCCQ (*Kansas City Cardiomyopathy Questionnaire*). L’utilisation d’inhibiteurs du SGLT-2 améliore le score sommaire global au questionnaire KCCQ, comparativement à un placebo (différence moyenne de 2,0, intervalle de confiance [IC] à 95 % de 1,6 à 2,5; certitude élevée). Un plus grand nombre de patients ayant reçu un inhibiteur du SGLT-2 ont obtenu une amélioration d’importance clinique de la QdV (rapport de risques de 1,14, IC à 95 % de 1,02 à 1,28; certitude modérée). Des améliorations similaires ont été observées pour le score sommaire clinique et le score des symptômes totaux du KCCQ, ainsi que dans tous les sous-groupes et à tous les moments de l’évaluation.

**Conclusions:**

L’utilisation d’un inhibiteur du SGLT-2 procure de manière constante une amélioration d’importance clinique de la QdV chez les patients atteints d’IC, indépendamment de la fraction d’éjection, des améliorations notables étant observées dès la deuxième semaine.

Heart failure (HF) is a multifactorial disease, and its global burden is rising and thus becoming a major cause of death, disability, and reduced quality of life (QoL). In Canada alone, 750,000 adults live with HF, and 100,000 are newly diagnosed with HF every year.^1^ Over the past decade, several therapies have emerged that improve outcomes of patients with HF.^2^^,^^3^ Among these, use of a sodium-glucose cotransporter-2 inhibitor (SGLT2i) has demonstrated improvements in both clinical outcomes, such as hospitalization and death, and QoL, in both clinical trials and previous meta-analyses conducted across the spectrum of left ventricular ejection fraction (LVEF), and regardless of setting.^4^^,^^5^ However, no comprehensive synthesis of the effect of SGLT2i use on QoL has been made, with regard to the effect of agent, LVEF category, and time period.^6^^,^^7^ HF patients with poor QoL often experience various physical and emotional symptoms, such as dyspnea, fatigue, depression, and sleeping difficulties.^8^ Whereas some effects of pharmacotherapy are delayed, interventions that provide rapid improvement in symptoms and QoL are valued by patients, yet few studies have explored the onset and durability of the QoL impact of SGLT2i use in HF. Additionally, studies have demonstrated some heterogeneity of treatment effect among different agents with SGLT2is in type 2 diabetes.^9^ This study aimed to assess the impact of use of an SGLT2i, compared with placebo, on QoL in patients with HF, and explore the consistency of effect, over time, among agents, and across the LVEF spectrum.

## Methods

We performed a systematic review and meta-analysis according to the 2020 Preferred Reporting of Systematic Reviews and Meta-Analyses (PRISMA) guidelines.^10^ The study protocol, registered *a priori*, is available on PROSPERO (CRD42022306201).

### Search strategy

Updating and adapting a previous systematic review from our group,^11^ we searched the Cochrane Central Register of Controlled Trials (CENTRAL), Embase, and MEDLINE, across the period from 2019 to February 2022, for published and ongoing studies. The search queries for all 3 databases are available in Supplemental Appendix S1.

### Eligibility criteria

We included studies that met all of the following eligibility criteria: (i) were parallel, placebo-controlled randomized controlled trials (RCTs) with blinding of participants, clinicians, and outcome assessors (“double-blind”); (ii) included patients with HF, irrespective of LVEF; (iii) compared an SGLT2i (eg, canagliflozin, dapagliflozin, empagliflozin, or other) or mixed SGLT1/2 inhibitor (eg, sotagliflozin) to placebo (with identical opportunity for all other cointerventions in both groups); (iv) reported HF-specific QoL (eg, 12-item or 23-item Kansas City Cardiomyopathy Questionnaire [KCCQ], Minnesota Living with Heart Failure Questionnaire [MLHFQ], or their subscores); reported in the English language.

We classified trials as including patients with HF with reduced (≤ 40%), mildly reduced (41%-49%), preserved (≥ 50%), or mixed LVEF, based on the 2021 Universal Definition and Classification of Heart Failure.^12^

### Outcomes

The primary outcome was the mean difference in QoL between the SGLT2i group and the placebo group at the furthest follow-up. The KCCQ is available as a 12-item or 23-item self-administered questionnaire that is used to quantify a patient’s health status along 6 different domains (physical functions, symptom frequency and burden, symptom stability, social limitations, QoL, and self-efficacy).^13^, ^14^, ^15^ Responses are summarized as a total score (ranging from 0 [worst] to 100 [best]), termed the overall summary score (OSS), as well as 2 subscores—the clinical summary score (CSS) and the total symptoms score (TSS). When multiple KCCQ subscores were reported, we extracted the OSS, the CSS, and the TSS.

The secondary outcome of interest was the difference between groups in the proportion of patients achieving a minimally important improvement in QoL (as defined in the original studies) at the furthest follow-up.

### Study selection and data extraction

One reviewer (R.D.T.) searched all 3 databases and imported the results into Covidence (Melbourne, Australia). Two reviewers (Y.R.S., R.D.T.) independently screened article titles and abstracts, and reviewed full texts for inclusion. One reviewer (Y.R.S.) extracted data using a standardized extraction form, and the second reviewer (R.D.T.) reviewed all data extraction for errors. The 2 reviewers resolved all disagreements by discussion.

The following variables were extracted: lead author last name; publication year; journal title; total sample size; number randomized to the SGLT2i and the control groups; trial duration; inclusion criteria (LVEF and New York Heart Association [NYHA] class); baseline characteristics (age, sex, NYHA class, prevalence of ischemic cardiomyopathy, prevalence of atrial fibrillation, LVEF, serum natriuretic peptide concentration); baseline HF medications; intervention characteristics (agent and dose); and outcome data. When trial reports noted having insufficient data for meta-analysis, we contacted the corresponding authors for additional data.

### Risk-of-bias and certainty-of-evidence assessment

Both reviewers independently assessed individual trials for risk of bias using the Cochrane Risk of Bias (RoB) 2 tool.^16^ In brief, we classified each trial as having a high, low, or unclear risk of bias, based on 5 domains, as follows: (i) a randomization process; (ii) deviations from intended interventions; (iii) missing outcome data; (iv) measurements of the outcome; and (v) selection of the reported result. We assessed for small-study effects, which may indicate publication bias, by assessing for funnel-plot asymmetry for the primary outcome. In cases in which visual asymmetry was observed, we used the trim-and-fill method to adjust for bias from potentially missing studies.^17^ The Grading of Recommendations Assessment, Development and Evaluation (GRADE) approach then was used to rate the certainty of evidence for each outcome of interest, using a minimally contextualized approach with a threshold of any difference between groups, based on risk of bias, inconsistency, imprecision, indirectness, and publication bias.^18^

### Data analysis

We presented the primary outcome as a mean difference, and the secondary outcome as a risk ratio (RR). We used a Mantel-Haenszel random-effects model, with a Paule-Mandel estimator and the Hartung-Knapp variance estimate, which provides more-conservative 95% confidence intervals (CIs) when heterogeneity is high, compared with the Dersemonian-Laird estimate,^19^^,^^20^ to calculate effect estimates with 95% CIs. For dichotomous outcomes, we then calculated the number needed to treat (NNT), as the reciprocal of the absolute risk differences. We evaluated statistical heterogeneity with a visual inspection of the forest plot and calculation of the I^2^ statistic.^21^

We performed several subgroup and sensitivity analyses on the primary outcome, as follows: (i) stratified and meta-regression analyses by timepoint ranging from 2 to 52 weeks; (ii) trial-level subgroup analysis based on trial-level LVEF classification, including reduced (≤ 40%) and mildly reduced (41%-49%) or preserved (≥ 50%) LVEF; (iii) trial-level subgroup analysis by agent, including canagliflozin, dapagliflozin, and empagliflozin. We performed all analyses using R version 4.0.5 (package: "meta"; R Foundation, Vienna, Austria). *P*-values < 0.05 were considered statistically significant.

## Results

### Study identification and key study characteristics

**Figure 1:**
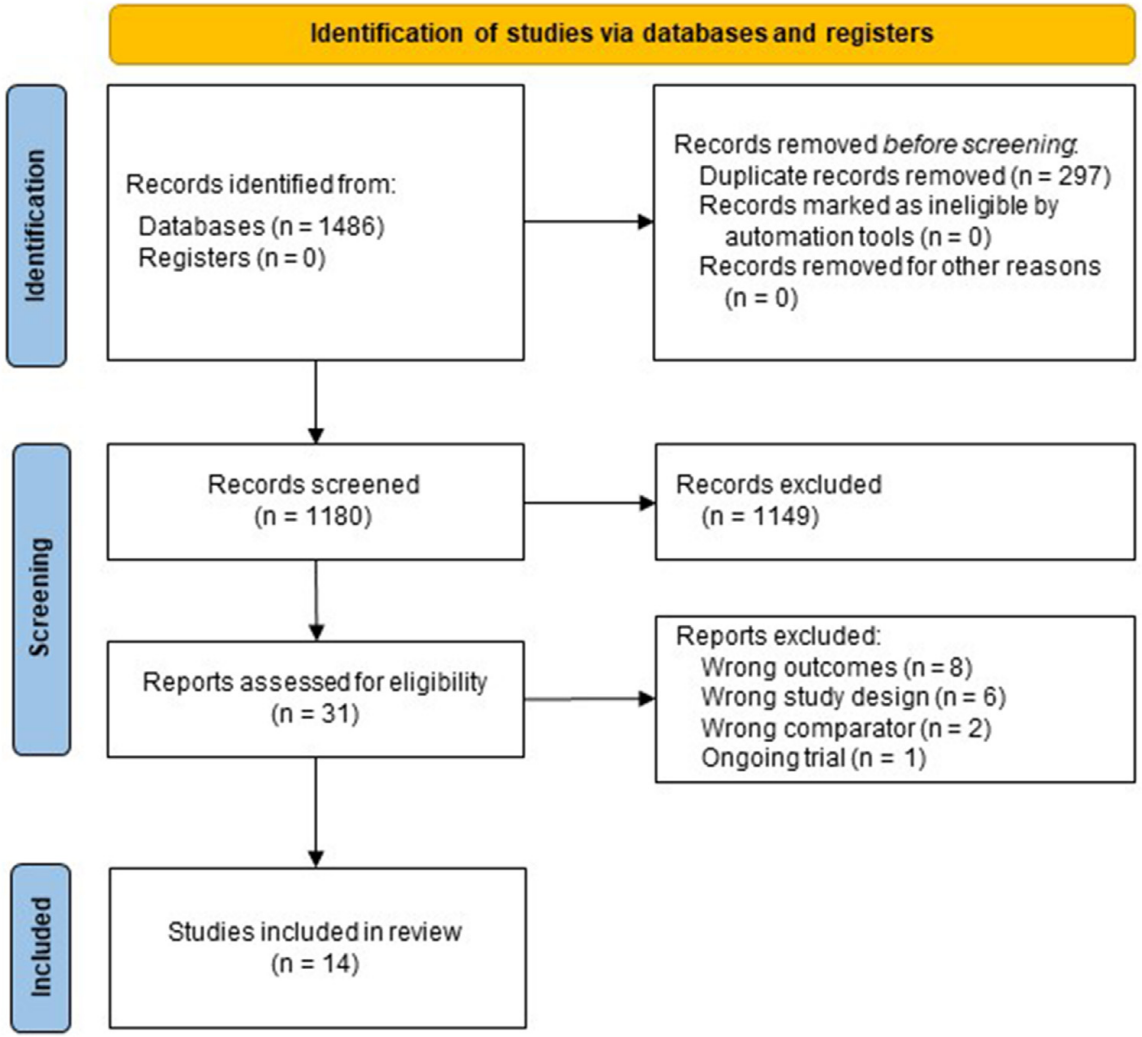
Preferred Reporting of Systematic Reviews and Meta-Analyses (PRISMA) 2020 study flow diagram.

Of the 1477 identified records, we included 14 RCTs (n = 23,361) that met our eligibility criteria (Fig. 1). We sought and obtained additional unpublished data for our primary outcome from the investigators of the **D**apagliflozin **a**nd **P**revention of **A**dverse Outcomes in **H**eart **F**ailure (DAPA-HF) and the **Emp**agliflozin 10 mg compared with placebo, initiated in Patients Hospitalized With Ac**u**te Heart Fai**l**ure Who Have Been **S**tabiliz**e**d (EMPULSE) trial. Details of the 17 articles excluded at full-text review are described in Supplemental Table S1. Across all 14 trials, the median age was 68 years, 34% of the participants were women, 67% of the patients were in NYHA functional class 2, and the median LVEF was 33% (Table 1). Six trials (n = 9344) enrolled exclusively patients with HF with reduced ejection fraction (HFrEF); 4 trials (n = 12,890) enrolled patients with HF and LVEF > 40%; and 4 trials (n = 1127) included patients with HF irrespective of LVEF. Canagliflozin was used in 1 trial (n = 448), dapagliflozin was used in 4 trials (n = 11,594), and empagliflozin was used in 9 trials (n = 11,319). All trials reported QoL as KCCQ-OSS or its subscores, with the timing of the QoL assessment ranging from 2 to 52 weeks, and the longest QoL follow-up median being 12 weeks (range: 12-52 weeks).

**Table 1:** Characteristics of included studies

Study	n	Age, y, mean	Women, %	NYHA class II/III/IV, %	LVEF, mean, %	Intervention	QoL instrument	Duration of follow-up, wk	Intervention group n/ control group n
CHIEF-HF	448	63.4	44.9	NR	NR	Canagliflozin	KCCQ	12	222/226
DAPA-HF	4744	66.4	23.4	67.5/31.6/0.91	31.1	Dapagliflozin	KCCQ	32	2373/2371
DEFINE-HF	263	61.3	27	66/34	26.5	Dapagliflozin	KCCQ	12	131/132
DELIVER	6263	71.7	43.9	75.3/24.5/0.3	54.2	Dapagliflozin	KCCQ	32	2903/2892
EMBRACE-HF	65	66	37	46/52.3/1.7	44	Empagliflozin	KCCQ	12	33/32
EMPA-TROPISM	84	62	36	NR	36	Empagliflozin	KCCQ	24	42/42
EMPERIAL-Preserved	315	73.5	43.2	77.1/22.5/0.4	53	Empagliflozin	KCCQ	12	157/158
EMPERIAL-Reduced	312	69	25.6	64.7/35.3	30	Empagliflozin	KCCQ	12	156/156
EMPEROR-Preserved	5988	71.9	44.7	81.5/18.1/0.3	54.3	Empagliflozin	KCCQ	52	2997/29,915
EMPEROR-Reduced	3730	66.9	24	75.1/24.4/0.6	27.5	Empagliflozin	KCCQ	52	1863/1867
EMPIRE-HF	190	64	15	78/16	29	Empagliflozin	KCCQ	12	95/95
EMPULSE	530	71	34	35.1/52.7/9.3	31.5	Empagliflozin	KCCQ	12	265/265
Preserved-HF	324	70	57	57	60	Dapagliflozin	KCCQ	12	162/162
SUGAR-DM-HF	105	68.7	26.7	77.1/22.9/0	32.5	Empagliflozin	KCCQ	36	52/53

CHIEF-HF, **C**anagliflozin Impact on **H**ealth Status, Quality of L**i**f**e** and **F**unctional Status in **H**eart **F**ailure; DAPA-HF, **D**apagliflozin **a**nd **P**revention of **A**dverse Outcomes in **H**eart **F**ailure; DEFINE-HF, **D**apagliflozin **Ef**fect on Symptoms and B**i**omarkers i**n** Pati**e**nts With **H**eart **F**ailure; DELIVER, **D**apagliflozin **E**valuation to Improve the **Live**s Patients With P**r**eserved Ejection Fraction Heart Failure; EMBRACE-HF, **Em**pagliflozin Evaluation **B**y Measu**r**ing Imp**a**ct on Hemodynami**e**s in Pati**e**nts with **H**eart **F**ailure; EMPA-TROPISM, Are the "Cardiac Benefits" of Empagliflozin Independent of Its Hypoglycemic Activity? (ATRU-4); EMPERIAL, Effect of **Emp**agliflozin on **E**xe**r**cise Ability and Heart Failure Symptoms **i**n Patients With Chronic He**a**rt Fai**l**ure; EMPEROR-Preserved, **Emp**agliflozin Outcom**e** T**r**ial in Patients With Chr**o**nic Hea**r**t Failure With **Preserved** Ejection Fraction; EMPEROR-Reduced, **Emp**agliflozin Outcom**e** T**r**ial in Patients With Chr**o**nic Hea**r**t Failure With **Reduced** Ejection Fraction; Empire-HF, Empagliflozin in Heart Failure Patients With Reduced Ejection Fraction; EMPULSE, **Emp**agliflozin in Patients Hospitalized With Ac**u**te Heart Fai**l**ure Who Have Been **S**tabiliz**e**d; KCCQ, Kansas City Cardiomyopathy Questionnaire; LVEF, left ventricular ejection fraction; NYHA, New York Heart Association; PRESERVED-HF, Dapagliflozin in **Preserved** Ejection Fraction **H**eart **F**ailure; QoL, quality of life; SUGAR-DM-HF, **S**t**u**dies of Empa**g**liflozin and Its C**a**rdiovascular, **R**enal and Metabolic Effects in Patients With **D**iabetes **M**ellitus (or Pre-diabetes) and **H**eart **F**ailure; NR, not reported.

### Risk of bias and certainty of evidence

We rated all 14 trials as being at low risk of bias in all domains (Supplemental Fig. S1), and we rated all outcomes as having high certainty. Table 2 provides a summary of the certainty of evidence and estimates of effect for all outcomes. Funnel plots for KCCQ-OSS and KCCQ-CSS, but not for KCCQ-TSS, indicated some asymmetry; however, adjusting for this asymmetry using the trim-and-fill method did not significantly change the study results (Supplemental Fig. S2).

**Table 2:** Grading of Recommendations Assessment, Development and Evaluation (GRADE) summary-of-findings table

KCCQ outcome	Certainty of evidence (sample size, n)	MD or RR (95% CI)	Absolute risk difference (95% CI)
OSS	High (19,074)	MD +2.05 (+1.52 to +2.56)	—
CSS	High (18,492)	MD +2.25 (+1.58 to +2.92)	—
TSS	High (18,814)	MD +2.32 (+1.73 to +2.92)	—
OSS responder	High (22,788)	RR 1.14 (1.02 to 1.28)	62 more per 1000 [NNT, 17] (from 9 to 124 more)
CSS responder	High (22,788)	RR 1.15 (1.04 to 1.27)	65 more per 1000 [NNT, 16] (from 17 to 117 more)
TSS responder	High (22,788)	RR 1.09 (1.06 to 1.12)	44 more per 1000 [NNT, 23] (from 29 to 59 more)

CI, confidence interval; CSS, Clinical Summary Score; KCCQ, Kansas City Cardiomyopathy Questionnaire; MD, mean difference; NNT, number needed to treat; OSS, Overall Summary Score; RR, relative risk; TSS, Total Symptom Score.

### Primary outcome: effect of SGLT2i use on health-related QoL at latest follow-up time

**Figure 2:**
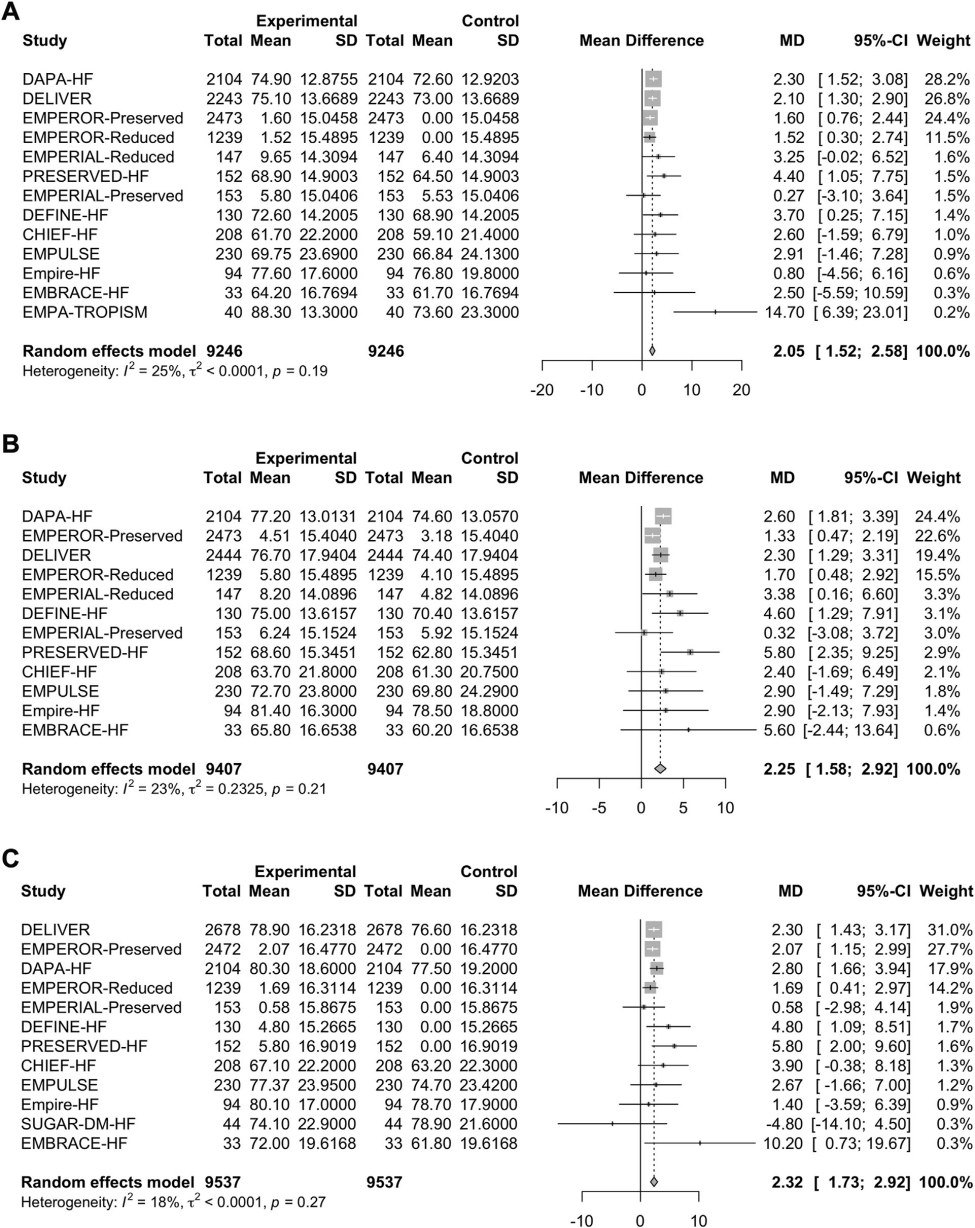
Forest plot of mean difference between sodium-glucose cotransporter-2 inhibitors (SGLT2i) and placebo in Kansas City Cardiomyopathy Questionnaire (KCCQ) (A) overall summary score, (B) clinical summary score, and (C) total symptoms score. KCCQ subscores show use of an SGLT2i providing a mean 2-point improvement in quality of life (QoL), compared to placebo. Heterogeneity is minimal, as shown by the I^2^ value of 25% or less and the visual overlap of the mean differences, meaning variability between trials is low in terms of the results of the trials. CHIEF-HF, **C**anagliflozin Impact on **H**ealth Status, Quality of L**i**f**e** and **F**unctional Status in **H**eart **F**ailure; CI, confidence interval; DAPA-HF, **D**apagliflozin **a**nd **P**revention of **A**dverse Outcomes in **H**eart **F**ailure; DEFINE-HF, **D**apagliflozin **Ef**fect on Symptoms and B**i**omarkers i**n** Pati**e**nts With **H**eart **F**ailure; DELIVER, **D**apagliflozin **E**valuation to Improve the **Live**s Patients With P**r**eserved Ejection Fraction Heart Failure; EMBRACE-HF, **Em**pagliflozin Evaluation **B**y Measu**r**ing Imp**a**ct on Hemodynami**e**s in Pati**e**nts with **H**eart **F**ailure; EMPA-TROPISM, Are the "Cardiac Benefits" of Empagliflozin Independent of Its Hypoglycemic Activity? (ATRU-4); EMPERIAL, Effect of **Emp**agliflozin on **E**xe**r**cise Ability and Heart Failure Symptoms **i**n Patients With Chronic He**a**rt Fai**l**ure; EMPEROR-Preserved, **Emp**agliflozin Outcom**e** T**r**ial in Patients With Chr**o**nic Hea**r**t Failure With **Preserved** Ejection Fraction; EMPEROR-Reduced, **Emp**agliflozin Outcom**e** T**r**ial in Patients With Chr**o**nic Hea**r**t Failure With **Reduced** Ejection Fraction; Empire-HF, Empagliflozin in Heart Failure Patients With Reduced Ejection Fraction; EMPULSE, **Emp**agliflozin in Patients Hospitalized With Ac**u**te Heart Fai**l**ure Who Have Been **S**tabiliz**e**d; MD, mean difference; PRESERVED-HF, Dapagliflozin in **Preserved** Ejection Fraction **H**eart **F**ailure; SD, standard deviation; SUGAR-DM-HF, Studies of Empagliflozin and Its Cardiovascular, Renal and Metabolic Effects in Patients With Diabetes Mellitus, or Prediabetes, and Heart Failure.

The mean difference in KCCQ-OSS was reported in 13 studies (n = 18,492). In the random-effects model, a significant improvement occurred in KCCQ-OSS with an SGLT2i, compared with placebo (mean difference 2.05, 95% CI 1.52-2.58; Fig. 2A). Heterogeneity was low, as indicated by an I^2^ value of 25%. Effects on the KCCQ-TSS and KCCQ-CSS were consistent with the KCCQ-OSS results (Table 2; Fig. 2, B and C).

### Sensitivity and subgroup analyses

**Figure 3:**
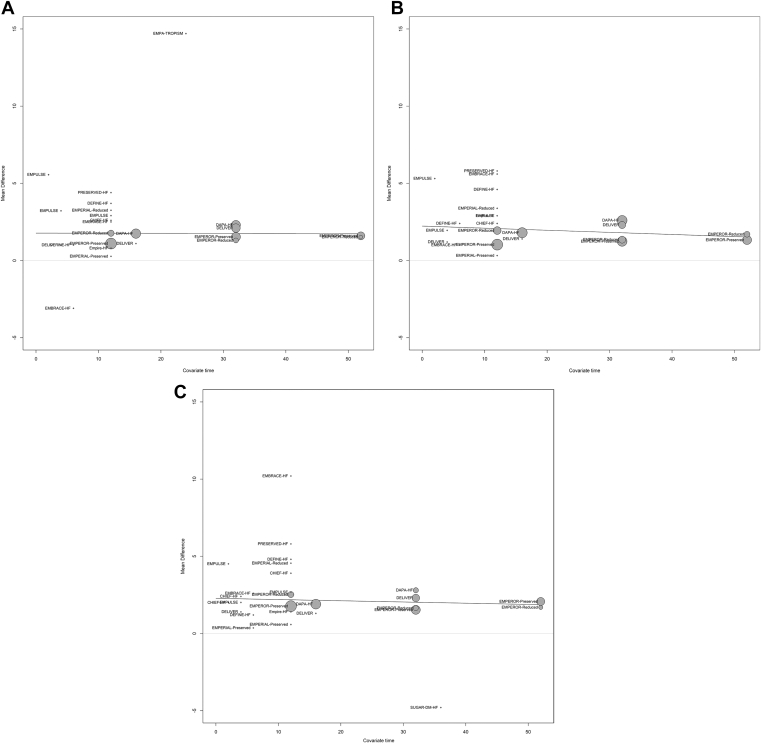
Meta-regression plots for mean difference between sodium-glucose cotransporter-2 (SGLT2) inhibitors and placebo over time for Kansas City Cardiomyopathy Questionnaire (**A**) overall summary score, (**B**) clinical summary score, and (**C**) total symptoms scale. CHIEF-HF, **C**anagliflozin Impact on **H**ealth Status, Quality of L**i**f**e** and **F**unctional Status in **H**eart **F**ailure; DAPA-HF, **D**apagliflozin **a**nd **P**revention of **A**dverse Outcomes in **H**eart **F**ailure; DEFINE-HF, **D**apagliflozin **Ef**fect on Symptoms and B**i**omarkers i**n** Pati**e**nts With **H**eart **F**ailure; DELIVER, **D**apagliflozin **E**valuation to Improve the **Live**s Patients With P**r**eserved Ejection Fraction Heart Failure; EMBRACE-HF, **Em**pagliflozin Evaluation **B**y Measu**r**ing Imp**a**ct on Hemodynami**e**s in Pati**e**nts with **H**eart **F**ailure; EMPA-TROPISM, Are the "Cardiac Benefits" of Empagliflozin Independent of Its Hypoglycemic Activity? (ATRU-4); EMPERIAL, Effect of **Emp**agliflozin on **E**xe**r**cise Ability and Heart Failure Symptoms **i**n Patients With Chronic He**a**rt Fai**l**ure; EMPEROR-Preserved, **Emp**agliflozin Outcom**e** T**r**ial in Patients With Chr**o**nic Hea**r**t Failure With **Preserved** Ejection Fraction; EMPEROR-Reduced, **Emp**agliflozin Outcom**e** T**r**ial in Patients With Chr**o**nic Hea**r**t Failure With **Reduced** Ejection Fraction; Empire-HF, Empagliflozin in Heart Failure Patients With Reduced Ejection Fraction; EMPULSE, **Emp**agliflozin in Patients Hospitalized With Ac**u**te Heart Fai**l**ure Who Have Been **S**tabiliz**e**d; MD, mean difference; PRESERVED-HF, Dapagliflozin in **Preserved** Ejection Fraction **H**eart **F**ailure; SD, standard deviation; SUGAR-DM-HF, **S**t**u**dies of Empa**g**liflozin and Its C**a**rdiovascular, **R**enal and Metabolic Effects in Patients With **D**iabetes **M**ellitus (or Pre-diabetes) and **H**eart **F**ailure.

Mean differences in KCCQ-OSS, KCCQ-CSS, and KCCQ-TSS were consistent over time in meta-regression, with no statistically significant moderator effect (*P* > 0.33 for all; Fig. 3). This consistency was also shown in a meta-analysis stratified by time (Table 3; Supplemental Fig. S3). Mean differences in KCCQ-OSS, KCCQ-CSS, and KCCQ-TSS were also consistent, regardless of the trial LVEF eligibility criteria (all *P*-values for interaction ≥ 0.3) and the SGLT2i used (all *P*-values for interaction > 0.05; Table 3; Supplemental Figs. S4 and S5).

**Table 3:** Subgroup analyses

Outcome	KCCQ-OSS		KCCQ-CSS		KCCQ-TSS	
	MD (95% CI)	Number of studies (sample size, n)	MD (95% CI)	Number of studies (sample size, n)	MD (95% CI)	Number of studies (sample size, n)
Timepoint	*P* = 0.04		*P* = 0.67		*P* = 0.82	
LVEF category, %	*P* = 0.30		*P* = 0.31		*P* = 0.53	
≤ 40	2.15 (1.53–2.78)	5 (7428)	2.47 (1.84–3.10)	5 (7428)	2.35 (1.53–3.17)	5 (7222)
> 40	1.89 (1.33–2.45)	4 (10,042)	1.83 (1.20–2.46)	4 (10,444)	2.24 (1.62–2.85)	4 (10,910)
Mixed	3.96 (1.28–6.65)	4 (1022)	2.99 (0.19–5.80)	3 (942)	3.94 (1.04–6.84)	3 (942)
Agent	*P* = 0.36		*P* = 0.06		*P* = 0.24	
Canagliflozin	2.60 (–1.59–6.79)	1 (416)	2.40 (–1.69–6.49)	1 (416)	3.90 (–0.38–8.18)	1 (416)
Dapagliflozin	2.30 (1.75–2.84)	4 (9258)	2.66 (2.06–3.26)	4 (9660)	2.66 (1.99–3.33)	4 (10,128)
Empagliflozin	1.69 (1.05–2.34)	8 (8818)	1.58 (0.92–2.23)	7 (8738)	1.90 (1.19–2.61)	7 (8530)

*P* values are for subgroup differences.

CI, confidence interval; CSS, Clinical Summary Score; KCCQ, Kansas City Cardiomyopathy Questionnaire; LVEF, left ventricular ejection fraction; MD, mean difference; OSS, Overall Summary Score; TSS, Total Symptom Score.

### Secondary outcome: effect of an SGLT2i on minimal important improvement in QoL

**Figure 4:**
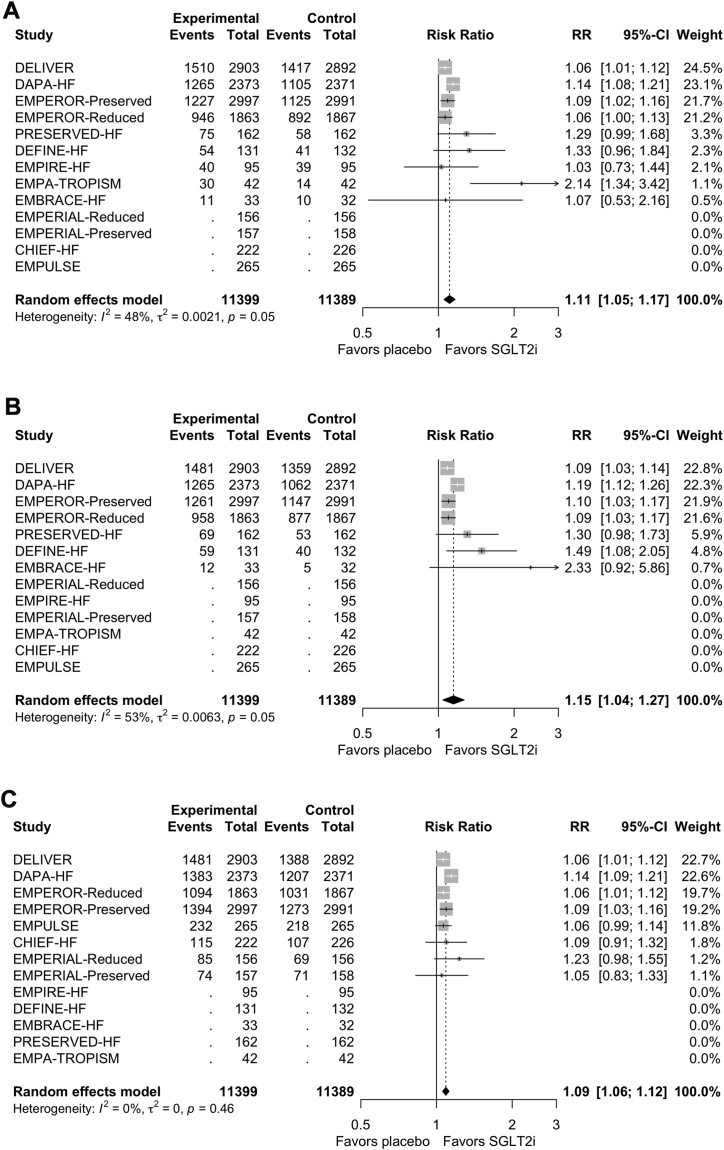
Forest plot for secondary outcome (proportion of patients with improvement greater than minimal important difference) for Kansas City Cardiomyopathy Questionnaire (KCCQ) (**A**) overall summary score, (**B**) clinical summary score, and (**C**) total symptoms scale. Risk ratios (RRs) for each KCCQ subscore were calculated for trials that provided a minimal important difference. The RR > 1 indicates that the incidence of patients experiencing a minimal improvement in quality of life was increased in the sodium-glucose cotransporter-2 inhibitors (SGLT2i) group, compared to the placebo group. Studies were weighed according to their sample size, with larger studies contributing more to the overall RR. CHIEF-HF, **C**anagliflozin Impact on **H**ealth Status, Quality of L**i**f**e** and **F**unctional Status in **H**eart **F**ailure; CI, confidence interval; DAPA-HF, **D**apagliflozin **a**nd **P**revention of **A**dverse Outcomes in **H**eart **F**ailure; DEFINE-HF, **D**apagliflozin **Ef**fect on Symptoms and B**i**omarkers i**n** Pati**e**nts With **H**eart **F**ailure; DELIVER, **D**apagliflozin **E**valuation to Improve the **Live**s Patients With P**r**eserved Ejection Fraction Heart Failure; EMBRACE-HF, **Em**pagliflozin Evaluation **B**y Measu**r**ing Imp**a**ct on Hemodynami**e**s in Pati**e**nts with **H**eart **F**ailure; EMPA-TROPISM, Are the "Cardiac Benefits" of Empagliflozin Independent of Its Hypoglycemic Activity? (ATRU-4); EMPERIAL, Effect of **Emp**agliflozin on **E**xe**r**cise Ability and Heart Failure Symptoms **i**n Patients With Chronic He**a**rt Fai**l**ure; EMPEROR-Preserved, **Emp**agliflozin Outcom**e** T**r**ial in Patients With Chr**o**nic Hea**r**t Failure With **Preserved** Ejection Fraction; EMPEROR-Reduced, **Emp**agliflozin Outcom**e** T**r**ial in Patients With Chr**o**nic Hea**r**t Failure With **Reduced** Ejection Fraction; Empire-HF, Empagliflozin in Heart Failure Patients With Reduced Ejection Fraction; EMPULSE, **Emp**agliflozin in Patients Hospitalized With Ac**u**te Heart Fai**l**ure Who Have Been **S**tabiliz**e**d; MD, mean difference; PRESERVED-HF, Dapagliflozin in **Preserved** Ejection Fraction **H**eart **F**ailure; SD, standard deviation; SUGAR-DM-HF, **S**t**u**dies of Empa**g**liflozin and Its C**a**rdiovascular, **R**enal and Metabolic Effects in Patients With **D**iabetes **M**ellitus (or Pre-diabetes) and **H**eart **F**ailure.

The clinically important improvement was defined in all included trials as a 5-point improvement or greater in the KCCQ score.^14^ The proportion of patients experiencing a clinically important improvement in KCCQ-OSS was reported in 9 studies (n = 22,788). Use of an SGLT2i significantly increased the probability of a clinically important improvement occurring in KCCQ-OSS (RR 1.14, 95% CI 1.02-1.28; I^2^ = 48%; absolute increase of 62 per 1000 [NNT, 17]; Fig. 4A). Heterogeneity was driven by a single outlier trial (Are the "Cardiac Benefits" of Empagliflozin Independent of Its Hypoglycemic Activity? [ATRU-4; EMPA-TROPISM]). Similarly, use of an SGLT2i increased the proportion of patients experiencing a clinically important improvement in KCCQ-CSS (RR 1.15, 95% CI 1.04-1.27; I^2^ = 53%; absolute increase of 65 per 1000 [NNT, 16]; Fig. 4B) and KCCQ-TSS (RR 1.09, 95% CI 1.06-1.12; I^2^ = 0%; absolute increase of 44 per 1000 [NNT, 23]; Fig. 4C).

## Discussion

In this systematic review and meta-analysis of RCTs, use of an SGLT2i provided clinically important improvements in QoL in patients with HF, based on high-certainty evidence. The QoL improvement from SGLT2i use was remarkably consistent, regardless of LVEF and which SGLT2i was used. Notably, the QoL impact of use of an SGLT2i can be noticed as early as week 2, and it persists to a similar extent at up to at least 1 year. Overall, at last available follow-up, of up to 1 year, results for the KCCQ overall summary score translated to an NNT of 17, for 1 patient with HF to experience a minimally important improvement in their QoL with use of an SGLT2i.^14^

These results extend the findings from individual RCTs and previous meta-analyses. We previously reported a comprehensive systematic review with meta-analysis of all RCTs reporting on the QoL impact of any pharmacotherapy for HFrEF.^11^ At the time, based on only 3 RCTs of 6877 participants, use of an SGLT2i demonstrated a clinically important improvement in QoL in HFrEF that was as good as or was greater than that of any other pharmacologic intervention, except for repletion of iron deficiency. Other studies also have been consistent with the present analyses. A 2021 meta-analysis by He et al. of RCTs of use of an SGLT2i in HFrEF showed a similar ∼2-point mean difference, compared with placebo, in the KCCQ-OSS.^22^ A 2022 meta-analysis by Yang et al. of use of an SGLT2i in HF with preserved ejection fraction similarly found an ∼2.7 mean difference in KCCQ-TSS with use of an SGLT2i vs placebo.^23^ In contrast to these studies, our meta-analysis included patients with both HFrEF and LVEF > 40%. HF with LVEF > 40% accounts for approximately 40% to 50% of incident HF^24^; thus, the present meta-analysis includes many patients that were excluded from these prior meta-analyses. Given the differential impact of SGLT2i use on certain outcomes in HF across the LVEF spectrum, most notably a mortality reduction in HFrEF but not in HFpEF, we explored for heterogeneity of treatment effect across the LVEF spectrum.^25^^,^^26^ Further, the present study included over 20-fold more patients compared to a 2022 meta-analysis by Guo et al.^27^ Moreover, the present analysis was the first to explore heterogeneity among different SGLT2is, or differences in their efficacy over time. These analyses demonstrated that QoL improvement in HF with use of SLGT2is is a class effect, with the onset of QoL improvement occurring as early as week 2, consistent with their rapid reduction in the risk of HF hospitalization within 18-30 days after initiation.^28^, ^29^, ^30^

The negative impact of HF on QoL is multifactorial, and improved QoL is an important goal for people living with this condition.^31^ Patients with HF often have strong preferences for either improving QoL or prolonging survival as their main priority.^6^^,^^32^, ^33^, ^34^, ^35^ Ideally, HF pharmacotherapy effectively achieves both goals, but this is not always the case.^11^ Although SGLT2i use improves survival only in patients with HFrEF, the impact of SGLT2i use on QoL and hospitalization is consistent and clinically important, regardless of LVEF. Use of an SGLT2i therefore represents a key intervention to offer to patients with HF in the form of shared decision-making, and the findings from the present study can be used to empower clinicians and patients in these discussions.

## Limitations

This systematic review with meta-analysis has some limitations that deserve consideration. First, included RCTs were inconsistent in the reporting of KCCQ subscores and in the timing of measurement, which led to having only a limited number of observations for certain timepoints (eg, 2-4 weeks). The observations at earlier timepoints, as a result, were imprecise due to the limited number of data points for KCCQ subscores, although they still demonstrated a statistically significant difference at 2 weeks. Second, the proportion of patients experiencing a clinically important improvement in KCCQ was reported inconsistently. Third, most included studies evaluated dapagliflozin or empagliflozin, and the ability to evaluate other agents is therefore limited. Finally, we did not identify any studies evaluating the effect of different doses of an SGLT2i on QoL in HF patients, and therefore, the dose-response relationship of these agents in HF remains unclear. Nevertheless, our study represents the most comprehensive review on the effect of SGLT2i use on QoL.

## Conclusion

SGLT2i use consistently provides a clinically important improvement in QoL among patients with HF, regardless of LVEF, with noticeable improvements seen as early as week 2. Clinicians can use these results to empower their patients with HF in shared decision-making.

## References

[1] Lawrence S. Heart failure in Canada: complex, incurable and on the rise. https://www.heartandstroke.ca/what-we-do/media-centre/news-releases/heart-failure-in-canada-complex-incurable-and-on-the-rise.

[2] McDonald M., Virani S., Chan M. (2021). CCS/CHFS heart failure guidelines update: defining a new pharmacologic standard of care for heart failure with reduced ejection fraction. Can J Cardiol.

[3] Behnoush A.H., Khalaji A., Naderi N., Ashraf H., von Haehling S. (2023). ACC/AHA/HFSA 2022 and ESC 2021 guidelines on heart failure comparison. ESC Heart Fail.

[4] Zou X., Shi Q., Vandvik P.O. (2022). Sodium-glucose cotransporter-2 inhibitors in patients with heart failure: a systematic review and meta-analysis. Ann Intern Med.

[5] Cardoso R., Graffunder F.P., Ternes C.M.P. (2021). SGLT2 inhibitors decrease cardiovascular death and heart failure hospitalizations in patients with heart failure: a systematic review and meta-analysis. EClinicalMedicine.

[6] Kraai I.H., Vermeulen K.M., Luttik M.L. (2013). Preferences of heart failure patients in daily clinical practice: quality of life or longevity?. Eur J Heart Fail.

[7] Lewis E.F., Johnson P.A., Johnson W. (2001). Preferences for quality of life or survival expressed by patients with heart failure. J Heart Lung Transplant.

[8] Heo S., Lennie T.A., Okoli C., Moser D.K. (2009). Quality of life in patients with heart failure: Ask the patients. Heart Lung.

[9] Jiang Y., Yang P., Fu L. (2022). Comparative cardiovascular outcomes of SGLT2 inhibitors in type 2 diabetes mellitus: a network meta-analysis of randomized controlled trials. Front Endocrinol (Lausanne).

[10] Page M.J., McKenzie J.E., Bossuyt P.M. (2021). The PRISMA 2020 statement: an updated guideline for reporting systematic reviews. BMJ.

[11] Turgeon R.D., Barry A.R., Hawkins N.M., Ellis U.M. (2021). Pharmacotherapy for heart failure with reduced ejection fraction and health-related quality of life: a systematic review and meta-analysis. Eur J Heart Fail.

[12] Bozkurt B., Coats A.J.S., Tsutsui H. (2021). Universal definition and classification of heart failure: a report of the Heart Failure Society of America, Heart Failure Association of the European Society of Cardiology, Japanese Heart Failure Society and Writing Committee of the Universal Definition of Heart Failure. J Card Fail.

[13] Garin O., Herdman M., Vilagut G. (2014). Assessing health-related quality of life in patients with heart failure: a systematic, standardized comparison of available measures. Heart Fail Rev.

[14] Kelkar A.A., Spertus J., Pang P. (2016). Utility of patient-reported outcome instruments in heart failure. JACC Heart Fail.

[15] Butler J., Khan M.S., Mori C. (2020). Minimal clinically important difference in quality of life scores for patients with heart failure and reduced ejection fraction. Eur J Heart Fail.

[16] Sterne J.A.C., Savovic J., Page M.J. (2019). RoB 2: a revised tool for assessing risk of bias in randomised trials. BMJ.

[17] Peters J.L., Sutton A.J., Jones D.R., Abrams K.R., Rushton L. (2007). Performance of the trim and fill method in the presence of publication bias and between-study heterogeneity. Stat Med.

[18] Balshem H., Helfand M., Schunemann H.J. (2011). GRADE guidelines: 3. Rating the quality of evidence. J Clin Epidemiol.

[19] Hartung J., Knapp G. (2001). A refined method for the meta-analysis of controlled clinical trials with binary outcome. Stat Med.

[20] Langan D., Higgins J.P.T., Jackson D. (2019). A comparison of heterogeneity variance estimators in simulated random-effects meta-analyses. Res Synth Methods.

[21] IntHout J., Ioannidis J.P.A., Rovers M.M., Goeman J.J. (2016). Plea for routinely presenting prediction intervals in meta-analysis. BMJ Open.

[22] He Z., Yang L., Nie Y. (2021). Effects of SGLT-2 inhibitors on health-related quality of life and exercise capacity in heart failure patients with reduced ejection fraction: a systematic review and meta-analysis. Int J Cardiol.

[23] Yang D., Zhang Y., Yan J., Liu M., An F. (2022). SGLT-2 inhibitors on prognosis and health-related quality of life in patients with heart failure and preserved ejection fraction: a systematic review and meta-analysis. Front Cardiovasc Med.

[24] Pfeffer M.A., Shah A.M., Borlaug B.A. (2019). Heart failure with preserved ejection fraction in perspective. Circ Res.

[25] Anker S.D., Butler J., Filippatos G. (2021). Empagliflozin in heart failure with a preserved ejection fraction. N Engl J Med.

[26] Solomon S.D., McMurray J.J.V., Claggett B. (2022). Dapagliflozin in heart failure with mildly reduced or preserved ejection fraction. N Engl J Med.

[27] Guo Z., Wang L., Yu J. (2023). The role of SGLT-2 inhibitors on health-related quality of life, exercise capacity, and volume depletion in patients with chronic heart failure: a meta-analysis of randomized controlled trials. Int J Clin Pharm.

[28] Berg D.D., Jhund P.S., Docherty K.F. (2021). Time to clinical benefit of dapagliflozin and significance of prior heart failure hospitalization in patients with heart failure with reduced ejection fraction. JAMA Cardiol.

[29] Vaduganathan M., Claggett B.L., Jhund P. (2022). Time to clinical benefit of dapagliflozin in patients with heart failure with mildly reduced or preserved ejection fraction: a prespecified secondary analysis of the DELIVER randomized clinical trial. JAMA Cardiol.

[30] Packer M., Butler J., Zannad F. (2021). Effect of empagliflozin on worsening heart failure events in patients with heart failure and preserved ejection fraction: EMPEROR-Preserved trial. Circulation.

[31] Berry C., McMurray J. (1999). A review of quality-of-life evaluations in patients with congestive heart failure. Pharmacoeconomics.

[32] MacDonald B.J., Barry A.R., Turgeon R.D. (2023). Decisional needs and patient treatment preferences for heart failure medications: a scoping review. CJC Open.

[33] Stevenson L.W., Hellkamp A.S., Leier C.V. (2008). Changing preferences for survival after hospitalization with advanced heart failure. J Am Coll Cardiol.

[34] Havranek E.P., McGovern K.M., Weinberger J. (1999). Patient preferences for heart failure treatment: utilities are valid measures of health-related quality of life in heart failure. J Card Fail.

[35] Brunner-La Rocca H.P., Rickenbacher P., Muzzarelli S. (2012). End-of-life preferences of elderly patients with chronic heart failure. Eur Heart J.

