# Supplementary material for: Impact of SGLT2 Inhibitors on Quality of Life in Heart Failure Across the Ejection Fraction Spectrum: Systematic Review and Meta-analysis
Source: CJC Open. 2023 Dec 10;6(4):639–48. doi: 10.1016/j.cjco.2023.12.002 (PMC11065673; doi:10.1016/j.cjco.2023.12.002)
Supplement: Supplementary Data [file mmc1.pdf]

Supplemental Appendix S1. Database search strategy

#	MEDLINE	Embase	CENTRAL
1	Heart Failure/	exp heart failure/	exp Heart Failure/
2	(cardiac failure* or heart failure* or myocardial failure*).mp.	(cardiac failure* or heart failure* or myocardial failure*).mp.	(cardiac failure* or heart failure* or myocardial failure*).mp. [mp=title, original title, abstract, mesh headings, heading words, keyword]
3	1 or 2	1 or 2	1 or 2
4	exp Sodium-Glucose Transporter 2 Inhibitors/	exp sodium glucose cotransporter 2 inhibitor/	(gliflozin* or sglt2* or SGLT-2* or sodium-glucose transport* 2* or sodium-glucose cotransport* 2* or canagliflozin* or Dapagliflozin* or Empagliflozin* or Ertugliflozin* or Ipragliflozin* or Luseogliflozin* or Remogliflozin etabonate* or Sotagliflozin* or Tofogliflozin*).mp.
5	(gliflozin* or sglt2* or SGLT-2* or sodium-glucose transport* 2* or sodium-glucose cotransport* 2* or canagliflozin* or Dapagliflozin* or Empagliflozin* or Ertugliflozin* or Ipragliflozin* or Luseogliflozin* or Remogliflozin etabonate* or Sotagliflozin* or Tofogliflozin*).mp.	(gliflozin* or sglt2* or SGLT-2* or sodium-glucose transport* 2* or sodium-glucose cotransport* 2* or canagliflozin* or Dapagliflozin* or Empagliflozin* or Ertugliflozin* or Ipragliflozin* or Luseogliflozin* or Remogliflozin etabonate* or Sotagliflozin* or Tofogliflozin*).mp.	3 and 4
6	4 or 5	4 or 5	quality of life.mp. or "Quality of Life"/
7	3 and 6	3 and 6	kansas city cardiomyopathy questionnaire.mp.
8	quality of life.mp. or "Quality of Life"/	quality of life.mp. or "Quality of Life"/	exp health status/

9	kansas city cardiomyopathy questionnaire.mp.	kansas city cardiomyopathy questionnaire.mp.	minnesota living with heart failure.mp.
10	Health Status/	exp health status/	kccq.mp.
11	minnesota living with heart failure.mp.	minnesota living with heart failure.mp.	lihfe.mp.
12	kccq.mp.	kccq.mp.	6 or 7 or 8 or 9 or 10 or 11
13	lihfe.mp.	lihfe.mp.	5 and 12
14	8 or 9 or 10 or 11 or 12 or 13	8 or 9 or 10 or 11 or 12 or 13	
15	limit 7 to "therapy (maximizes specificity)"	limit 7 to "therapy (maximizes specificity)"	
16	7 and 14	7 and 14	
17	15 or 16	15 or 16	
18	-	systematic review.ti.	
19	-	meta-analysis.ti.	
20	-	18 or 19	
21	-	17 not 20	

Supplemental Table S1: Studies Excluded After Full-Text Review

Study Identifier	Reason for exclusion
Akasaka H, Sugimoto K, Shintani A, Taniuchi S, Yamamoto K, Iwakura K, et al. Effects of ipragliflozin on left ventricular diastolic function in patients with type 2 diabetes and heart failure with preserved ejection fraction: The EXCEED randomized controlled multicenter study. Geriatr Gerontol Int. 2022;22(4):298-304.	No quality of life outcomes
Bonora BM, Vigili de Kreutzenberg S, Avogaro A, Fadini GP. Effects of the SGLT2 inhibitor dapagliflozin on cardiac function evaluated by impedance cardiography in patients with type 2 diabetes. Secondary analysis of a randomized placebo-controlled trial. Cardiovasc Diabetol. 2019;18(1):106.	No quality of life outcomes
Chambergo-Michilot D, Tauma-Arrue A, Loli-Guevara S. Effects and safety of SGLT2 inhibitors compared to placebo in patients with heart failure: A systematic review and meta-analysis. Int J Cardiol Heart Vasc. 2021;32:100690.	Wrong study design (systematic review)
Ejiri K, Miyoshi T, Kihara H, Hata Y, Nagano T, Takaishi A, et al. Drug effect of luseogliflozin and voglibose on heart failure with preserved ejection fraction in diabetic patients: a multicenter randomized-controlled trial. European Heart Journal. 2019;40(Supplement_1).	Wrong comparator (not placebo)
Ejiri K, Miyoshi T, Kihara H, Hata Y, Nagano T, Takaishi A, et al. Effect of Luseogliflozin on Heart Failure With Preserved Ejection Fraction in Patients With Diabetes Mellitus. Journal of the American Heart Association. 2020;9(16):e015103.	No quality of life outcomes
Moller J, Foundation DH, Herlev, Hospital G, Bispebjerg UH, Frederiksberg, et al. The Cardiac Effects of Empagliflozin in Patients With High Risk of Heart Failure. https://ClinicalTrials.gov/show/NCT05084235; 2021.	Ongoing trial
He Z, Yang L, Nie Y, Wang Y, Wang Y, Niu X, et al. Effects of SGLT-2 inhibitors on health-related quality of life and exercise capacity in heart failure patients with reduced ejection fraction: A systematic review and meta-analysis. Int J Cardiol. 2021;345:83-8.	Wrong study design (systematic review)
Ilyas F, Jones L, Tee SL, Horsfall M, Swan A, Wollaston F, et al. Acute pleiotropic effects of dapagliflozin in type 2 diabetic patients with heart failure with reduced ejection fraction: a crossover trial. ESC Heart Fail. 2021;8(5):4346-52.	No quality of life outcomes, wrong design (crossover trial)
Jensen J, Omar M, Kistorp C, Poulsen MK, Tuxen C, Gustafsson I, et al. Empagliflozin in heart failure patients with reduced ejection fraction: a randomized clinical trial (Empire HF). Trials. 2019;20(1):374.	Trial protocol
Kolwelter J, Bosch A, Jung S, Stabel L, Kannenkeril D, Ott C, et al. Effects of the sodium-glucose cotransporter 2 inhibitor empagliflozin on vascular function in patients with chronic heart failure. ESC Heart Fail. 2021;8(6):5327-37.	No quality of life outcomes

McEwan P, McMurray JJV, Jhund PS, Docherty KF, Qin L. Evaluating the key predictors of health-related quality of life in patients with heart failure and reduced ejection fraction: results from the DAPA-HF trial. European Heart Journal. 2021;42(Supplement_1).	Wrong study design (different DAPA-HF analysis included in the meta-analysis)
Nunez J, Palau P, Dominguez E, Mollar A, Nunez E, Ramon JM, et al. Early effects of empagliflozin on exercise tolerance in patients with heart failure: A pilot study. Clin Cardiol. 2018;41(4):476-80.	Wrong study design
Omar M, Jensen J, Frederiksen PH, Kistorp C, Videbaek L, Poulsen MK, et al. Effect of Empagliflozin on Hemodynamics in Patients With Heart Failure and Reduced Ejection Fraction. J Am Coll Cardiol. 2020;76(23):2740-51.	No quality of life outcomes
Singh, et al. Research into the effect of sodium-glucose linked transporter inhibition in left ventricular remodelling in patients with heart failure and diabetes mellitus (REFORM Trial). Scottish Medical Journal. 2019;64(4):NP64-NP5.	Wrong outcomes
Tamaki S, Yamada T, Watanabe T, Morita T, Furukawa Y, Kawasaki M, et al. Effect of Empagliflozin as an Add-On Therapy on Decongestion and Renal Function in Patients With Diabetes Hospitalized for Acute Decompensated Heart Failure: A Prospective Randomized Controlled Study. Circ Heart Fail. 2021;14(3):e007048.	No quality of life outcomes
Tanaka A, Hisauchi I, Taguchi I, Sezai A, Toyoda S, Sata M, et al. Abstract 9549: Effects of Canagliflozin in Patients With Type 2 Diabetes and Chronic Heart Failure: A Randomized Clinical Trial (CANDLE). Circulation. 2019;140(Suppl_1):A9549-A.<br>Tanaka A, Hisauchi I, Taguchi I, Sezai A, Toyoda S, Tomiyama H, et al. Effects of canagliflozin in patients with type 2 diabetes and chronic heart failure: a randomized trial (CANDLE). European Heart Journal. 2020;41(Supplement_2).	Wrong comparator (not placebo)

Supplemental Figure S1

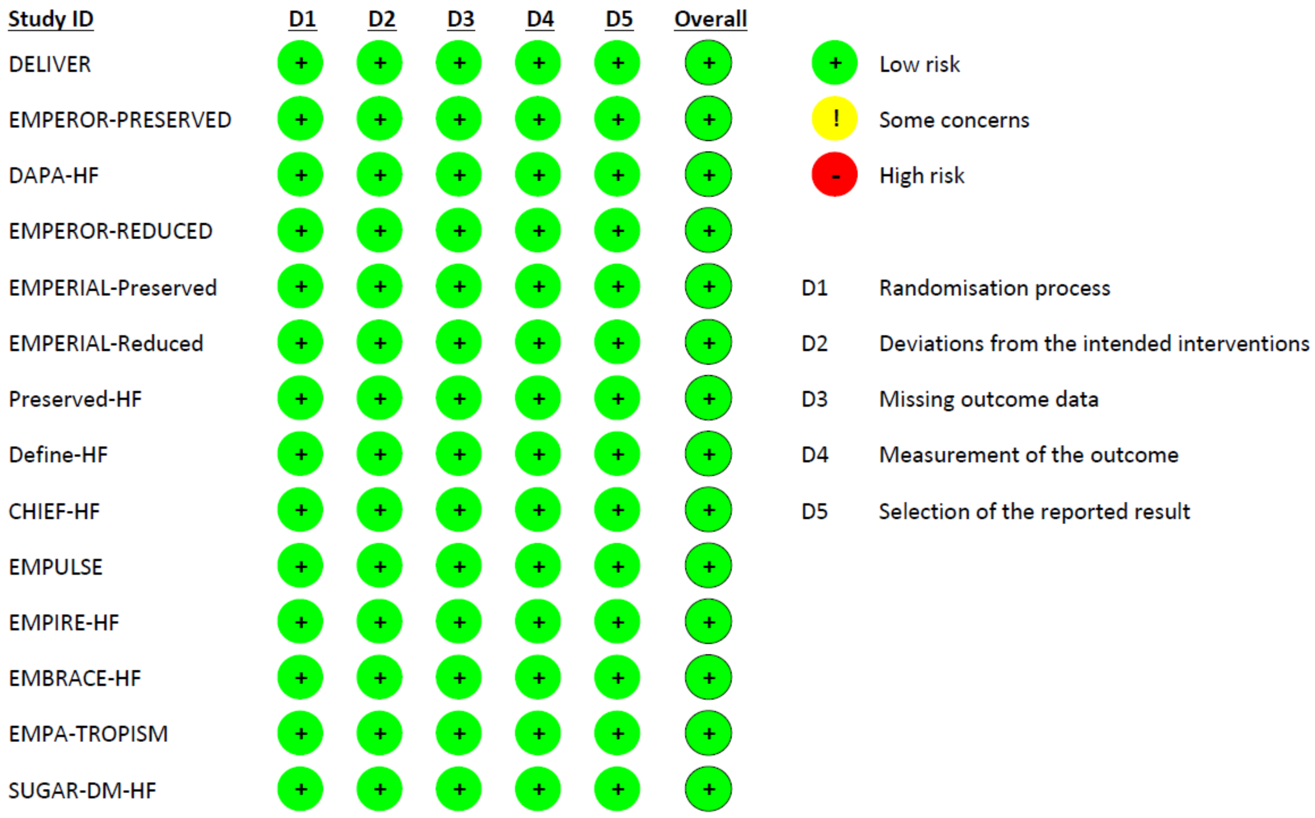

Supplemental Figure S2: Funnel plots for (A) KCCQ-OSS, (B) KCCQ-CSS, and (C) KCCQ-TSS and results of the trim-and-fill analysis accounting for potential missing studies.

(A) KCCQ-OSS

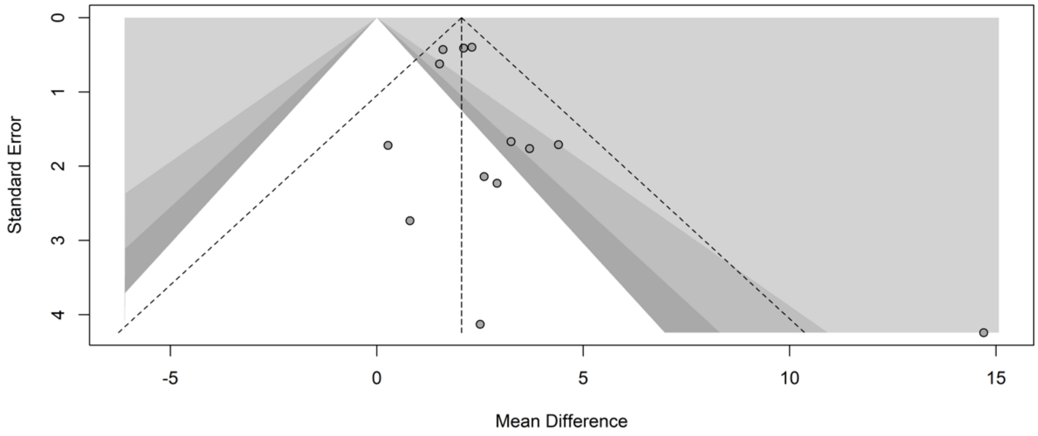

Estimate by trim-and-fill with 3 added studies: Mean difference 1.96 (95% confidence interval 1.35-2.57) [primary analysis 2.05, 1.52-2.56]

(B) KCCQ-CSS

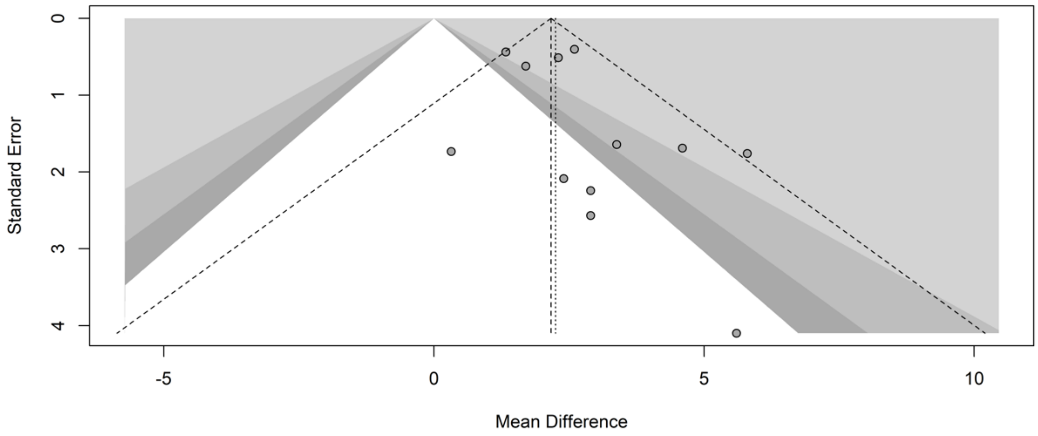

Estimate by trim-and-fill with 4 added studies: Mean difference 2.00 (95% confidence interval 1.27-2.73) [primary analysis 2.25, 1.58-2.92]

(C) KCCQ-TSS

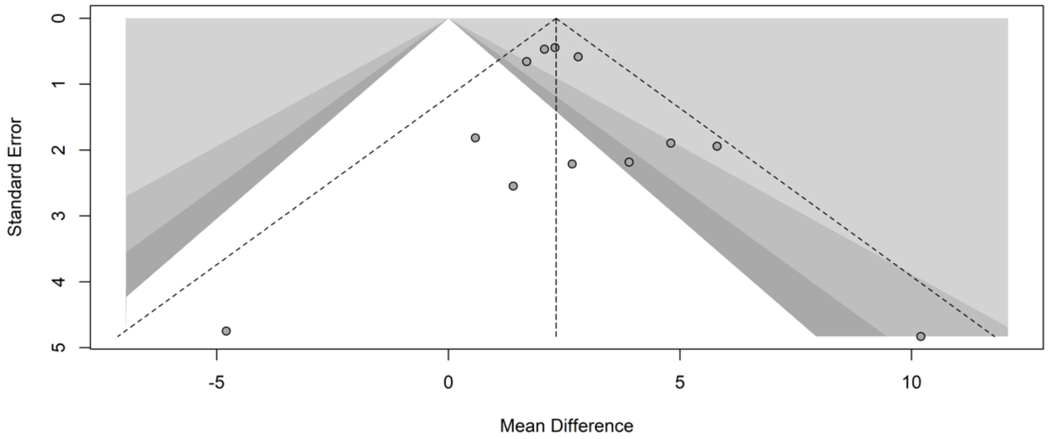

Estimate by trim-and-fill with 1 added study: Mean difference 2.30 (95% confidence interval 1.68-2.92) [primary analysis 2.32, 1.73-2.92]

Supplemental Figure S3: Meta-analysis of the impact of sodium-glucose co-transporter 2 inhibitors (SGLT2i) on HRQoL compared to placebo, stratified by time-point. (A) KCCQ-OSS (B) KCCQ-CSS (C) KCCQ-TSS scores.

(A) KCCQ-OSS

	Experimental			Control				
Study	Total	Mean	SD	Total	Mean	SD	MD	95%-CI
time = 2								
EMPULSE	233	64.33	23.1800	233	58.77	23.7900	5.56	[1.30; 9.82]
time = 4								
DELIVER	2678	72.50	.	2678	71.50	.	1.00	
EMPULSE	237	66.92	24.1500	237	63.70	24.7200	3.22	[-1.18; 7.62]
time = 6								
DEFINE-HF	130	71.10	14.4082	130	70.10	14.4082	1.00	[-2.50; 4.50]
EMBRACE-HF	33	63.50	16.8110	33	66.60	16.8110	-3.10	[-11.21; 5.01]
Common effect model	163			163			0.36	[-2.86; 3.57]
Heterogeneity: $I^2 = 0\%$, $\tau^2 = 0$, $p = 0.36$								
time = 12								
CHIEF-HF	208	61.70	22.2000	208	59.10	21.4000	2.60	[-1.59; 6.79]
DEFINE-HF	130	72.60	14.2005	130	68.90	14.2005	3.70	[0.25; 7.15]
EMBRACE-HF	33	64.20	16.7694	33	61.70	16.7694	2.50	[-5.59; 10.59]
EMPERIAL-Preserved	153	5.80	15.0406	153	5.53	15.0406	0.27	[-3.10; 3.64]
EMPERIAL-Reduced	147	9.65	14.3094	147	6.40	14.3094	3.25	[-0.02; 6.52]
EMPEROR-Preserved	2846	1.10	13.5338	2846	0.00	13.5338	1.10	[0.40; 1.80]
EMPEROR-Reduced	1755	1.77	14.4611	1755	0.00	14.4611	1.77	[0.81; 2.73]
Empire-HF	94	77.60	17.6000	94	76.80	19.8000	0.80	[-4.56; 6.16]
EMPULSE	230	69.75	23.6900	230	66.84	24.1300	2.91	[-1.46; 7.28]
PRESERVED-HF	152	68.90	14.9003	152	64.50	14.9003	4.40	[1.05; 7.75]
Common effect model	5748			5748			1.53	[1.01; 2.06]
Heterogeneity: $I^2 = 0\%$, $\tau^2 = 0.2346$, $p = 0.50$								
time = 16								
DAPA-HF	2133	74.88	12.6822	2133	73.13	12.7338	1.74	[0.98; 2.51]
DELIVER	2444	73.90	.	2444	72.80	.	1.10	
time = 24								
EMPA-TROPISM	40	88.30	13.3000	40	73.60	23.3000	14.70	[6.39; 23.01]
time = 32								
DAPA-HF	2104	74.90	12.8755	2104	72.60	12.9203	2.30	[1.52; 3.08]
DELIVER	2243	75.10	13.6689	2243	73.00	13.6689	2.10	[1.30; 2.90]
EMPEROR-Preserved	2616	1.53	14.4291	2616	0.00	14.4291	1.53	[0.75; 2.31]
EMPEROR-Reduced	1618	1.30	15.5492	1618	0.00	15.5492	1.30	[0.23; 2.37]
Common effect model	8581			8581			1.87	[1.45; 2.29]
Heterogeneity: $I^2 = 9\%$, $\tau^2 = 0.0145$, $p = 0.35$								
time = 52								
EMPEROR-Preserved	2473	1.60	15.0458	2473	0.00	15.0458	1.60	[0.76; 2.44]
EMPEROR-Reduced	1239	1.52	15.4895	1239	0.00	15.4895	1.52	[0.30; 2.74]
Common effect model	3712			3712			1.57	[0.88; 2.27]
Heterogeneity: $I^2 = 0\%$, $\tau^2 = 0$, $p = 0.92$								

Heterogeneity: $I^2 = 23\%$, $\tau^2 = 0.0130$, $p = 0.16$

Test for subgroup differences: $\chi^2_7 = 14.78$, df = 7 ($p = 0.04$)

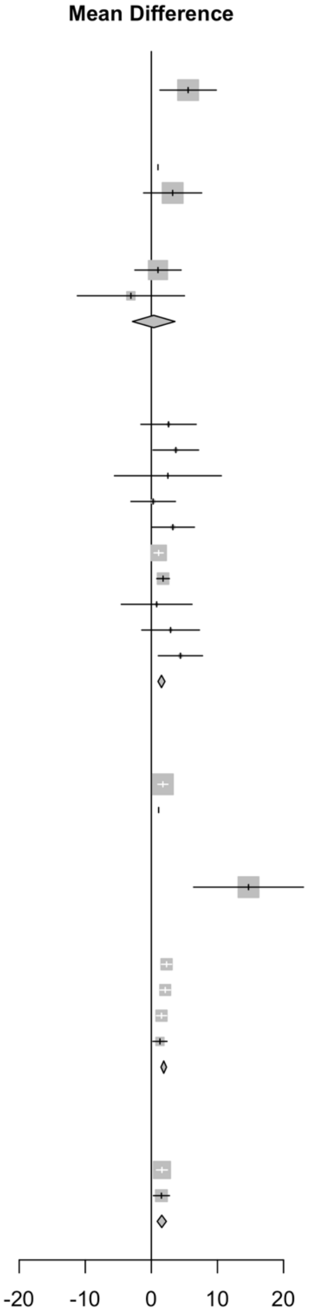

(B) KCCQ-CSS

Study	Experimental Total	Experimental Mean	Experimental SD	Control Total	Control Mean	Control SD	MD	95%-CI
**time = 2**								
EMPULSE	233	68.91	23.3600	233	63.60	23.7300	5.31	[1.03; 9.59]
**time = 4**								
DELIVER	2678	74.50	.	2678	73.30	.	1.20	
EMPULSE	237	70.00	24.6200	237	68.05	24.3200	1.95	[-2.46; 6.36]
**time = 6**								
DEFINE-HF	130	75.20	13.4498	130	72.80	13.4498	2.40	[-0.87; 5.67]
EMBRACE-HF	33	66.60	16.6965	33	65.60	16.6965	1.00	[-7.06; 9.06]
**Common effect model**	**163**			**163**			**2.20**	**[-0.83; 5.23]**
Heterogeneity: $I^2 = 0\%$, $\tau^2 = 0$, $p = 0.75$								
**time = 12**								
CHIEF-HF	208	63.70	21.8000	208	61.30	20.7500	2.40	[-1.69; 6.49]
DEFINE-HF	130	75.00	13.6157	130	70.40	13.6157	4.60	[1.29; 7.91]
EMBRACE-HF	33	65.80	16.6538	33	60.20	16.6538	5.60	[-2.44; 13.64]
EMPERIAL-Preserved	153	6.24	15.1524	153	5.92	15.1524	0.32	[-3.08; 3.72]
EMPERIAL-Reduced	147	8.20	14.0896	147	4.82	14.0896	3.38	[0.16; 6.60]
EMPEROR-Preserved	2864	1.03	13.6512	2864	0.00	13.6512	1.03	[0.32; 1.74]
EMPEROR-Reduced	1755	1.94	14.8377	1755	0.00	14.8377	1.94	[0.96; 2.92]
Empire-HF	94	81.40	16.3000	94	78.50	18.8000	2.90	[-2.13; 7.93]
EMPULSE	230	72.70	23.8000	230	69.80	24.2900	2.90	[-1.49; 7.29]
PRESERVED-HF	152	68.60	15.3451	152	62.80	15.3451	5.80	[2.35; 9.25]
**Common effect model**	**5766**			**5766**			**1.64**	**[1.11; 2.17]**
Heterogeneity: $I^2 = 41\%$, $\tau^2 = 1.1860$, $p = 0.09$								
**time = 16**								
DAPA-HF	2133	77.20	12.8162	2133	75.40	12.8675	1.80	[1.03; 2.57]
DELIVER	2444	75.70	.	2444	74.30	.	1.40	
**time = 32**								
DAPA-HF	2104	77.20	13.0131	2104	74.60	13.0570	2.60	[1.81; 3.39]
DELIVER	2243	76.70	17.9404	2243	74.40	17.9404	2.30	[1.25; 3.35]
EMPEROR-Preserved	2616	1.24	14.7048	2616	0.00	14.7048	1.24	[0.44; 2.04]
EMPEROR-Reduced	1618	1.35	15.4052	1618	0.00	15.4052	1.35	[0.29; 2.41]
**Common effect model**	**8581**			**8581**			**1.89**	**[1.44; 2.34]**
Heterogeneity: $I^2 = 59\%$, $\tau^2 = 0.2907$, $p = 0.06$								
**time = 52**								
EMPEROR-Preserved	2473	4.51	15.4040	2473	3.18	15.4040	1.33	[0.47; 2.19]
EMPEROR-Reduced	1239	5.80	15.4895	1239	4.10	15.4895	1.70	[0.48; 2.92]
**Common effect model**	**3712**			**3712**			**1.45**	**[0.75; 2.15]**
Heterogeneity: $I^2 = 0\%$, $\tau^2 = 0$, $p = 0.63$								

Heterogeneity: $I^2 = 25\%$, $\tau^2 = 0.1618$, $p = 0.14$

Test for subgroup differences: $\chi^2_6 = 4.03$, df = 6 ($p = 0.67$)

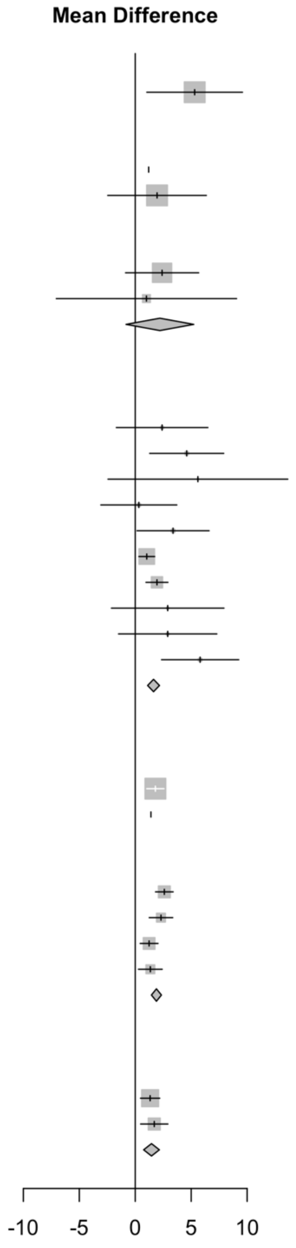

(**C) KCCQ-TSS**

Study	Experimental Total	Experimental Mean	Experimental SD	Control Total	Control Mean	Control SD	MD	95%-CI
**time = 2**								
CHIEF-HF	222	63.50	20.9000	222	61.50	21.2000	2.00	[-1.92; 5.92]
EMPULSE	233	72.94	23.1900	233	68.43	23.5700	4.51	[0.26; 8.76]
**Common effect model**	**455**			**455**			**3.15**	**[0.28; 6.03]**
Heterogeneity: $I^2 = 0\%$, $\tau^2 = 0$, $p = 0.39$								
**time = 4**								
CHIEF-HF	216	64.50	21.0000	216	62.10	21.5000	2.40	[-1.61; 6.41]
DELIVER	2678	77.10	.	2678	75.70	.	1.40	
EMPULSE	237	74.62	24.8500	237	72.61	24.2400	2.01	[-2.41; 6.43]
**Common effect model**	**3131**			**3131**			**2.22**	**[-0.74; 5.19]**
Heterogeneity: $I^2 = 0\%$, $\tau^2 = 0$, $p = 0.90$								
**time = 6**								
DEFINE-HF	130	1.20	15.6201	130	0.00	15.6201	1.20	[-2.60; 5.00]
EMBRACE-HF	33	70.10	17.8878	33	67.50	17.8878	2.60	[-6.03; 11.23]
EMPERIAL-Preserved	155	5.59	.	155	5.23	.	0.36	
EMPERIAL-Reduced	152	.	.	152	.	.		
**Common effect model**	**470**			**470**			**1.43**	**[-2.05; 4.90]**
Heterogeneity: $I^2 = 0\%$, $\tau^2 = 0$, $p = 0.77$								
**time = 12**								
CHIEF-HF	208	67.10	22.2000	208	63.20	22.3000	3.90	[-0.38; 8.18]
DEFINE-HF	130	4.80	15.2665	130	0.00	15.2665	4.80	[1.09; 8.51]
EMBRACE-HF	33	72.00	19.6168	33	61.80	19.6168	10.20	[0.73; 19.67]
EMPERIAL-Preserved	153	0.58	15.8675	153	0.00	15.8675	0.58	[-2.98; 4.14]
EMPERIAL-Reduced	147	4.55	14.8369	147	0.00	14.8369	4.55	[1.16; 7.94]
EMPEROR-Preserved	2846	1.77	15.1656	2846	0.00	15.1656	1.77	[0.98; 2.56]
EMPEROR-Reduced	1755	2.52	16.0428	1755	0.00	16.0428	2.52	[1.46; 3.58]
Empire-HF	94	80.10	17.0000	94	78.70	17.9000	1.40	[-3.59; 6.39]
EMPULSE	230	77.37	23.9500	230	74.70	23.4200	2.67	[-1.66; 7.00]
PRESERVED-HF	152	5.80	16.9019	152	0.00	16.9019	5.80	[2.00; 9.60]
**Common effect model**	**5748**			**5748**			**2.29**	**[1.71; 2.88]**
Heterogeneity: $I^2 = 30\%$, $\tau^2 = 0.5762$, $p = 0.17$								
**time = 16**								
DAPA-HF	2133	80.10	13.8461	2133	78.20	13.9044	1.90	[1.07; 2.73]
DELIVER	2444	77.90	.	2444	76.60	.	1.30	
**time = 32**								
DAPA-HF	2104	80.30	18.6000	2104	77.50	19.2000	2.80	[1.66; 3.94]
DELIVER	2243	78.90	16.2318	2243	76.60	16.2318	2.30	[1.35; 3.25]
EMPEROR-Preserved	2616	1.53	16.0818	2616	0.00	16.0818	1.53	[0.66; 2.40]
EMPEROR-Reduced	1618	1.64	16.6290	1618	0.00	16.6290	1.64	[0.49; 2.79]
**Common effect model**	**8581**			**8581**			**2.01**	**[1.51; 2.52]**
Heterogeneity: $I^2 = 20\%$, $\tau^2 = 0.0655$, $p = 0.29$								
**time = 36**								
SUGAR-DM-HF	44	74.10	22.9000	44	78.90	21.6000	-4.80	[-14.10; 4.50]
**time = 52**								
EMPEROR-Preserved	2472	2.07	16.4770	2472	0.00	16.4770	2.07	[1.15; 2.99]
EMPEROR-Reduced	1239	1.69	16.3114	1239	0.00	16.3114	1.69	[0.41; 2.97]
**Common effect model**	**3711**			**3711**			**1.94**	**[1.19; 2.69]**
Heterogeneity: $I^2 = 0\%$, $\tau^2 = 0$, $p = 0.64$								

Heterogeneity: $I^2 = 0\%$, $\tau^2 < 0.0001$, $p = 0.56$

Test for subgroup differences: $\chi^2_7 = 3.68$, df = 7 ($p = 0.82$)

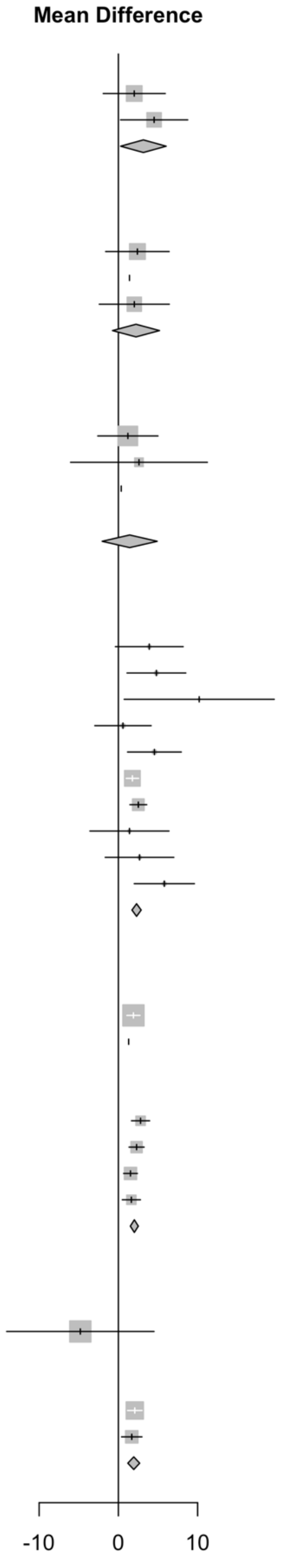

CI, confidence interval; SD, standard deviation; MD, mean difference.

Supplemental Figure S4: Meta-analysis of the impact of sodium-glucose co-transporter 2 inhibitors (SGLT2i) on HRQoL compared to placebo, stratified by ejection fraction category. (A) KCCQ-OSS, (B) KCCQ-CSS, (C) KCCQ-TSS scores.

(A) KCCQ-OSS

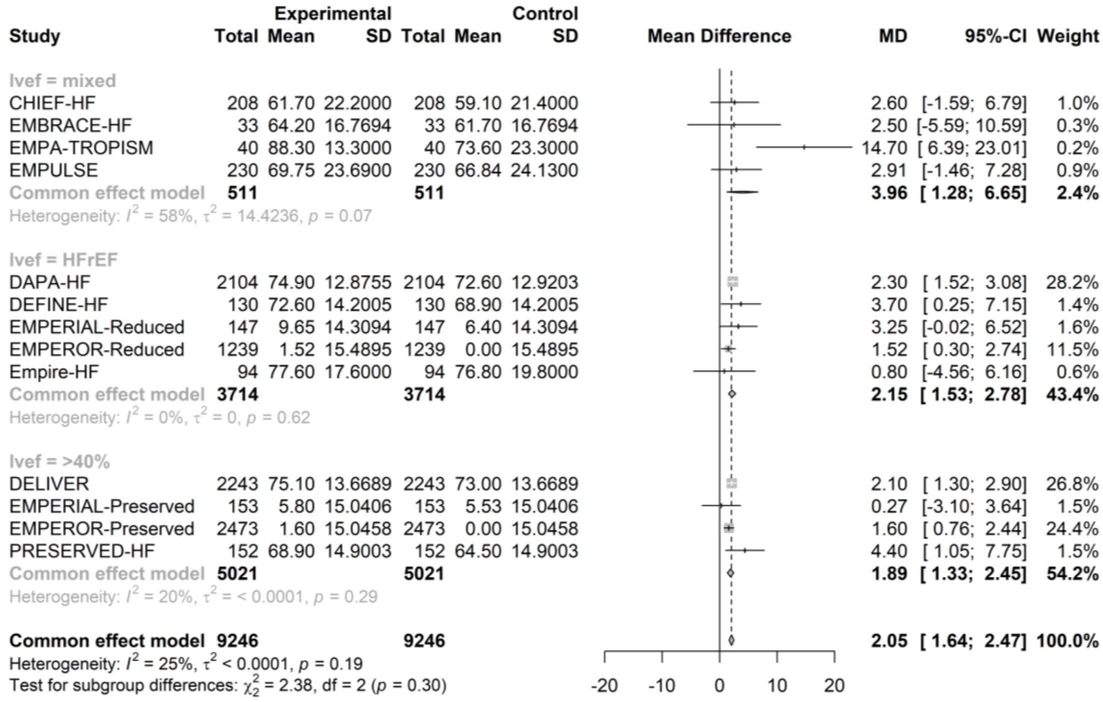

(B) KCCQ-CSS

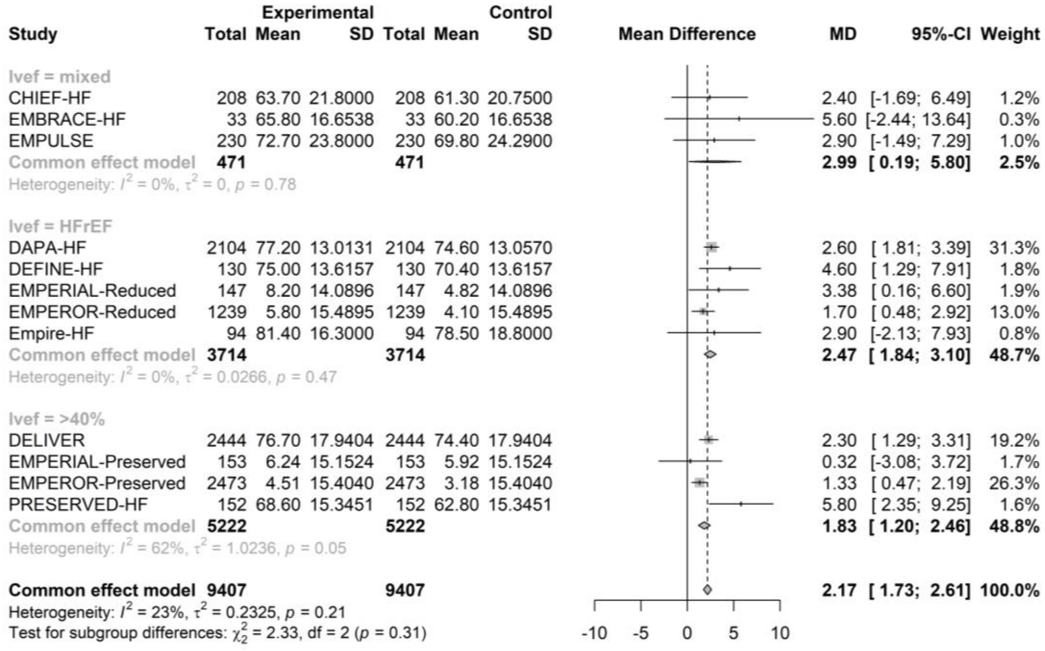

(C) KCCQ-TSS

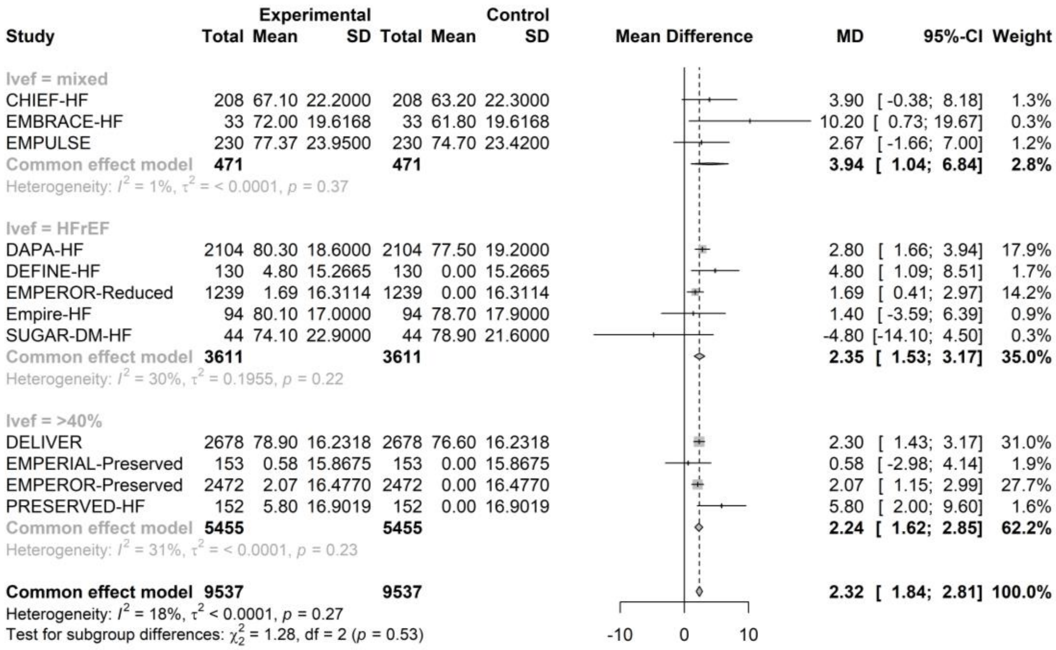

CI, confidence interval; SD, standard deviation; MD, mean difference.

Supplemental Figure S5: Meta-analysis of the impact of sodium-glucose co-transporter 2 inhibitors (SGLT2i) on HRQoL compared to placebo, stratified by agent. (A) KCCQ-OSS, (B) KCCQ-CSS, (C) KCCQ-TSS scores.

(A) KCCQ-OSS

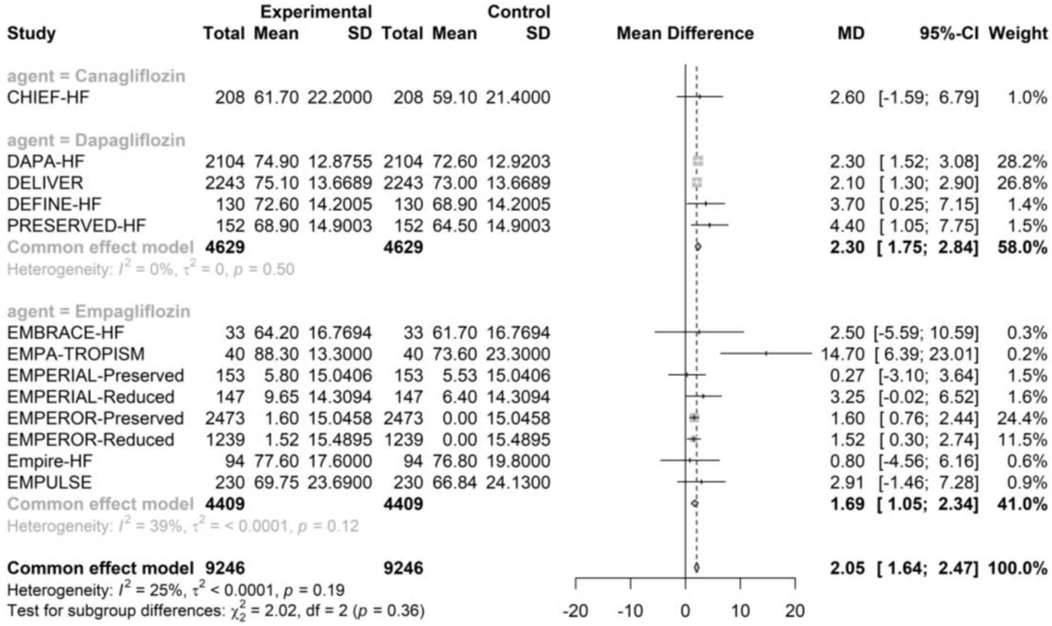

(B) KCCQ-CSS

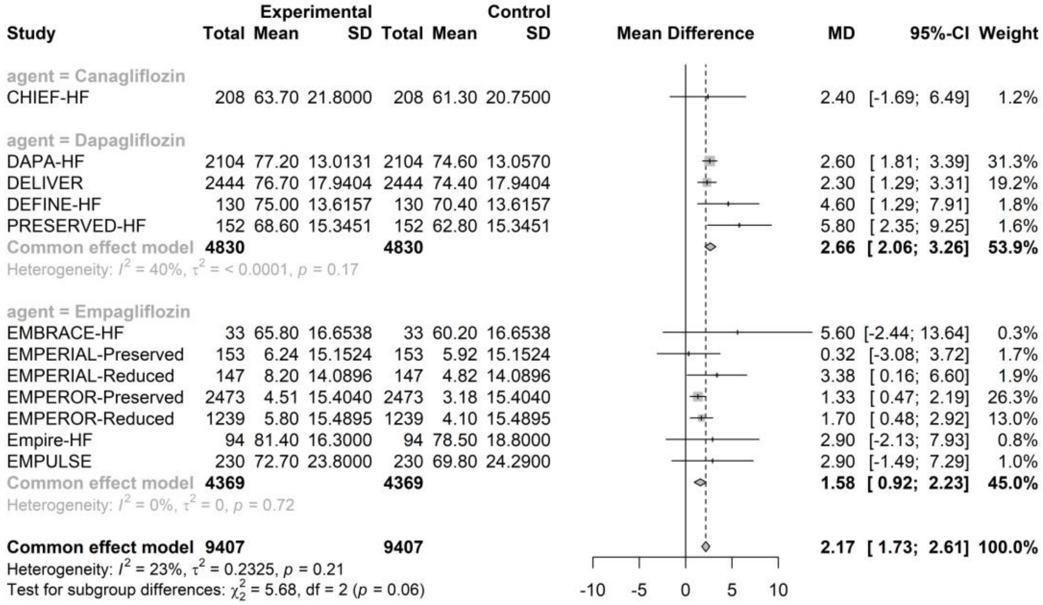

(C) KCCQ-TSS

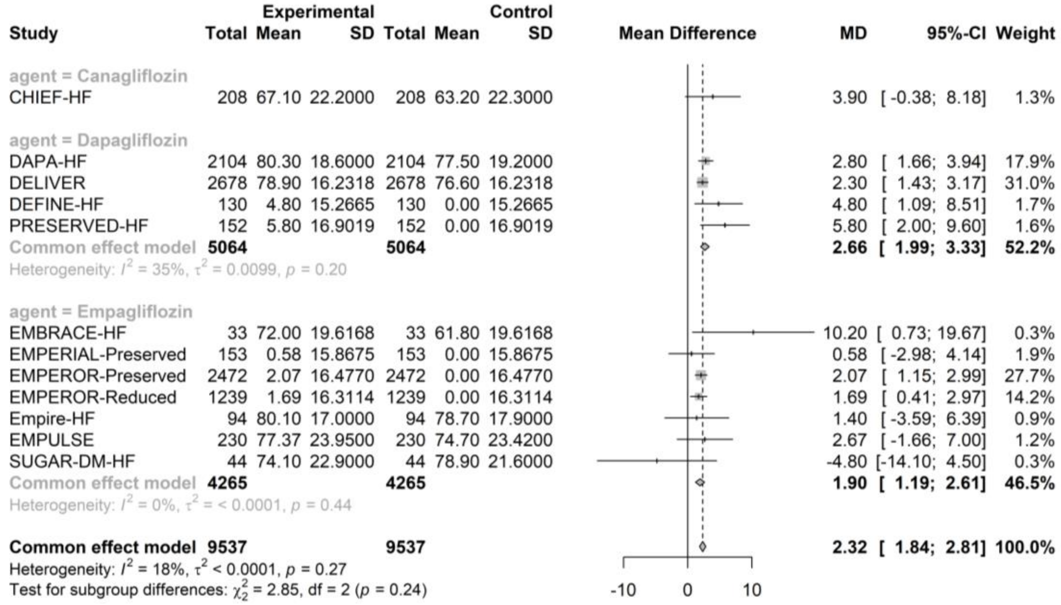

CI, confidence interval; SD, standard deviation; MD, mean difference.
